# Musculoskeletal health state and physical function of intensive care unit survivors: protocol for a UK multicentre prospective cohort study (the MSK-ICU study)

**DOI:** 10.1136/bmjopen-2022-071385

**Published:** 2023-02-02

**Authors:** Owen Gustafson, Elizabeth King, Michael Schlussel, Matthew Rowland, Helen Dawes, Mark A Williams

**Affiliations:** 1 Centre for Movement, Occupational and Rehabilitation Sciences (MOReS), Oxford Institute of Nursing, Midwifery and Allied Health Research (OxINMAHR), Faculty of Health and Life Sciences, Oxford Brookes University, Oxford, UK; 2 Oxford Allied Health Professions Research & Innovation Unit, Oxford University Hospitals NHS Foundation Trust, Oxford, UK; 3 Centre for Statistics in Medicine, Nuffield Department of Orthopaedics Rheumatology and Musculoskeletal Sciences, University of Oxford, Oxford, UK; 4 Oxford Critical Care Unit, Oxford University Hospitals NHS Foundation Trust, Oxford, UK; 5 Medical School, University of Exeter, Exeter, UK; 6 Exeter Biomedical Research Centre, Medical School, Faculty of Health and Life Sciences, University of Exeter, Exeter, UK

**Keywords:** adult intensive & critical care, rehabilitation medicine, musculoskeletal disorders

## Abstract

**Introduction:**

Survivors of critical illness frequently experience long-term physical impairment, decreased health-related quality of life and low rates of return to employment. There has been limited investigation of the underlying problems affecting physical function post-intensive care unit (ICU) admission. Musculoskeletal (MSK) conditions may be complex in presentation, with ICU survivors potentially at greater risk of their development due to the rapid muscle mass loss seen in ICU. The MSK health state of ICU survivors and its impact on physical function remain largely unknown. The aim of the MSK-ICU study is to determine and characterise the MSK health state of ICU survivors 6 months following admission to ICU, in order to inform development of targeted rehabilitation interventions.

**Methods and analysis:**

The MSK-ICU study is a multicentre prospective longitudinal cohort study, evaluating the MSK health state of ICU survivors 6 months after admission to ICU. The study consists of a primary study and two substudies. The primary study will be a telephone follow-up of adults admitted to ICU for more than 48 hours, collecting data on MSK health state, quality of life, employment, anxiety and depression and symptoms of post-traumatic stress disorder. The planned sample size is 334 participants. Multivariable regression will be used to identify prognostic factors for a worse MSK health state, as measured by the MSK-Health Questionnaire. In substudy 1, participants who self-report any MSK problem will undergo a detailed, in-person MSK physical assessment of pain, peripheral joint range of movement and strength. In substudy 2, participants reporting a severe MSK problem will undergo a detailed physical assessment of mobility, function and muscle architecture.

**Ethics and dissemination:**

Ethical approval has been obtained through the North of Scotland Research Ethics Committee 2 (21/NS/0143). We aim to disseminate the findings through international conferences, international peer-reviewed journals and social media.

**Trial registration number:**

ISRCTN24998809.

**Strengths and limitations of this study**

- The exploratory sub studies will provide rich evaluation of musculoskeletal structure and function.
- Bias through loss to follow-up will be minimised through a comprehensive participant retention plan.
- The study does not undertake a qualitative evaluation of patient experience which is recommended as part of complex intervention development.

## Introduction

The number of admissions to intensive care units (ICUs), complexity of illness and cost of critical care is increasing. This is representative of both an ageing critical care population presenting with a variety of pre-existing comorbidities and multimorbidity, and an increase in survival rates due to improvements in ICU services and delivery.^1^ Survivors of critical illness frequently experience long-term physical impairment, persistent exercise limitation and decreased health-related quality of life (QoL).^2^ The subsequent individual and socioeconomic burden of critical illness is high, with significant healthcare utilisation after discharge from hospital.^3^ Rates of return to employment following admission to ICU are also low, with up to 31% of patients not returning to work within five years of ICU admission.^4^

Musculoskeletal (MSK) conditions are wide ranging and include problems affecting bone, muscle and joints. They are the leading cause of pain and disability in the UK with 25% of the population affected.^5^ They are characterised by pain and loss of function and can diminish QoL and impact on family and social relationships.^5^ MSK conditions also have a significant socioeconomic impact. They are the second leading cause of sickness absence at work, with 30.8 million working days lost in the UK in 2016 due to MSK problems.^6^

Given the rates of muscle mass loss of up to 20% in the first week of ICU admission,^7^ it is reasonable to expect that patients will subsequently present with MSK complications after discharge from ICU. Therefore, it is possible that long-term MSK complications are contributing to poor physical function, QoL and return to work in ICU survivors.

Multiple recent studies investigating rehabilitation interventions after ICU and hospital discharge have failed to demonstrate positive primary outcomes for patient-reported physical function and exercise capacity.^8–10^ The interventions employed in these studies are based on the successful group exercise programmes used in cardiac and pulmonary rehabilitation,^11^ constituting cardiopulmonary and general strengthening exercises. It is unclear to what extent general weakness and decreased exercise capacity contribute to poor outcomes and thus explanatory power for the lack of effectiveness of these interventions. If other physical problems are found to influence function and QoL, more effective rehabilitation interventions could be designed and evaluated.

This potential source of long-term disability in ICU survivors is under-investigated. A scoping review of MSK complications following critical illness highlighted a number of studies investigating MSK health after hospital discharge.^12^ Most studies evaluated a single aspect of MSK health, with peripheral muscle weakness, chronic pain and abnormal neuromuscular function being the most commonly assessed and reported problems. A high prevalence of MSK complications were reported with the shoulder identified as the most commonly affected joint.^13 14^ To date no studies have evaluated the overall MSK health state of ICU survivors using MSK-specific patient-reported measures and work metrics.^15^

Considering the high prevalence of MSK conditions in ICU survivors, the unknown impact on physical function and QoL and the ineffectiveness of current post-ICU rehabilitation interventions, we propose a need to comprehensively evaluate the MSK and wider biopsychosocial systems of ICU survivors.

## Methods and analysis

### Aim and objectives

The aim of the MSK-ICU study is to determine and characterise the MSK health state of ICU survivors six months following admission to ICU.

Specifically, the study intends to:

1. Quantify the MSK health state using the MSK Health Questionnaire (MSK-HQ) and assess its relationship with QoL, employment, anxiety and depression and symptoms of post-traumatic stress disorder.
2. Identify prognostic factors for a lower MSK-HQ Score after critical illness.
3. Characterise the specific MSK complications experienced by patients using a standardised comprehensive MSK assessment.
4. Evaluate patient mobility and upper limb function, and the extent of the relationship to muscle structure and function in those patients with poor MSK health state.

### Study design

**Figure 1:**
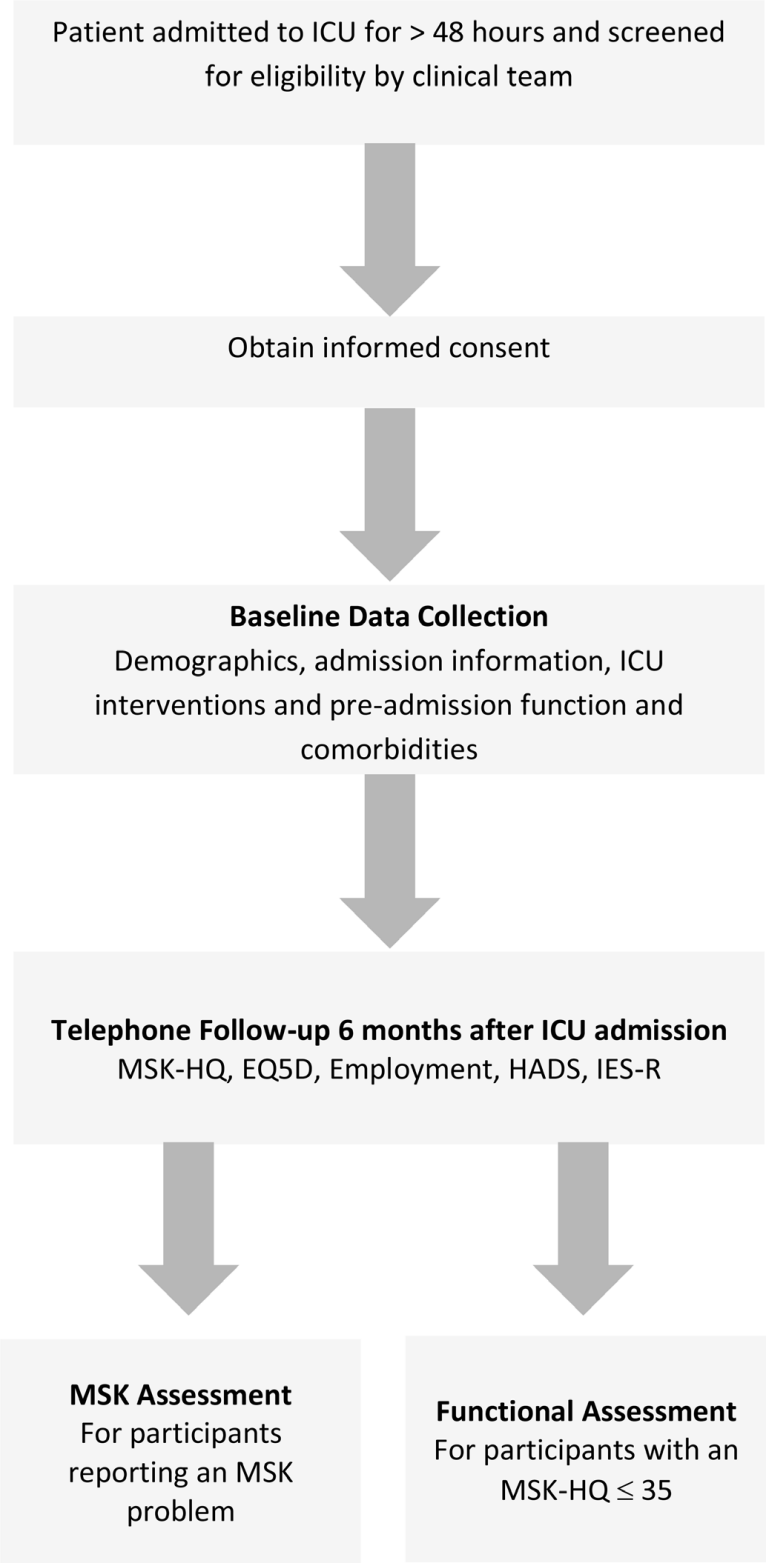
Study process. EQ5D, European Quality of Life Five Dimensions; HADS, Hospital Anxiety and Depression Score; ICU, intensive care unit; IES-R, Impact of Events Scale-Revised; MSK, musculoskeletal; MSK-HQ, Musculoskeletal Health Questionnaire.

The MSK-ICU study is a multicentre, prospective, longitudinal cohort study exploring the MSK health state of ICU survivors. Data collection is split into a primary study using a telephone follow-up questionnaire, and two substudies involving in-person assessments (see figure 1 for a representation of the flow of participants). Planned study start of recruitment is 18 February 2022 and is due to complete follow-up on 31 October 2023.

### Patient and public involvement

A patient and public advisory group was formed during the development of this study. The group was consulted on the design of the study with a focus on the structure of the telephone follow-up and in-person assessments, and the burden of participating. Members of this group are members of the external advisory committee, who will meet during the study to discuss the conduct of the study and the results.

### Study settings

The primary study and substudies will take place in four separate UK NHS trusts. There are approximately 1700 patients admitted for more than 48 hours and subsequently discharged from the general adult ICUs across the four trusts annually.

### Participant selection

#### Primary study: telephone follow-up

Patients aged 18 years or above admitted to one of the participating ICUs for more than 48 hours will be eligible for inclusion. Patients will be excluded if any of the following apply: proven or suspected primary brain pathology, spinal cord injury or other neuromuscular disease resulting in permanent or prolonged weakness; admitted to the ICU with MSK complications or trauma; a palliative diagnosis/treatment pathway; dependent for activities of daily living in the month prior to current ICU admission; no fixed abode; prisoner; unable to communicate clearly in English over the telephone for 20 min.

#### Substudy 1: in-person MSK assessment

Participants in the primary study who self-report any MSK problem will be invited to participate in substudy 1.

#### Substudy 2: in-person functional assessment

Participants in the primary study who report a severe MSK problem, as defined by an MSK-HQ Score of 35 or less, will be invited to participate in substudy 2.

### Consent

Patients will only be approached for consent if the clinical and research team deem that the patient has capacity to consent. Consultee consent will not be sought from patient’s next of kin. Written versions of the participant information sheet and informed consent will be presented to the patients, who will be given time to ask questions and consider the information. Written informed consent will then be obtained by means of participant-dated signature and dated signature of the researcher who presented and obtained the informed consent. Participants will be approached prior to leaving hospital.

Participants who are eligible for either substudy will be invited to participate in one of these at the telephone follow-up. Verbal versions of the relevant participant information will be presented to the participants. If the participant provisionally agrees to participate, a written version of the participant information will be posted or emailed to them. They will then be booked an appointment to attend an in-person assessment, and asked to contact the research team to cancel that appointment if they no longer wish to participate or wish to have more time to consider the information. The participant will have at least 48 hours between telephone follow-up and the in-person assessment appointment. At the assessment appointment, written informed consent will be confirmed and recorded.

### Study procedures

#### Primary study: telephone follow-up

The primary study will involve a single telephone follow-up at six months following admission to ICU. Baseline data collected for all enrolled participants will include: demographics (age, ethnicity, sex, body mass index); admission information (reason for admission, severity of illness, ICU and hospital length of stay); ICU interventions (invasive ventilation and duration, neuromuscular blocking agents, steroids, prone positioning, mobilisation activity); and preadmission function and comorbidities (Functional Comorbidity Index,^16^ Clinical Frailty Scale 17 and MSK history).

Prior to the six-month telephone call, patients’ electronic health record will be checked for their current health status and location that is, in hospital, at home or died. At the start of the phone call, participants will be asked if it is a convenient time to talk and that the questionnaires will take approximately 20 min. If it is not a convenient time, the researcher will arrange to call the participant back at their best convenience. Participants will then be asked five questionnaires in the following order: Musculoskeletal Health Questionnaire (MSK-HQ),^18^ European Quality of Life Five Dimensions (EQ-5D-5L),^19^ employment questionnaire,^3^ Hospital Anxiety and Depression Score (HADS)^20^ and Impact of Events Scale-Revised (IES-R).^21^ The EQ-5D-5L, HADS and IES-R form the core outcome set recommended for ICU follow-up studies.^22^

#### Substudy 1: in-person MSK assessment

Participants will be assessed at either their home or their local ICU follow-up clinic. Participants will undergo a three part MSK assessment. First participants will be asked to record their current pain severity and location using a visual analogue scale (VAS) and body map, prior to being asked the Fear-Avoidance Belief Questionnaire (FABQ)^23^ and Douleur Neuropathique 4 Questions questionnaire.^24^ Second, participants’ upper and lower limb range of movement (ROM) will be assessed. Finally, participant strength will be assessed using the Medical Research Council Sum Score (MRCSS)^25^ and hand held dynamometry.^26^

#### Substudy 2: in-person functional assessment

Participants will be assessed in The Movement Science Laboratory at Oxford Brookes University. Participants will answer a series of questions regarding their physical health before undergoing the same MSK assessment as substudy 1. Participants will then undergo a series of physical assessments. Cross-sectional area of rectus femoris^27^ and biceps brachii^28^ will be measured using ultrasound (US) prior to isokinetic dynamometry (IKD) measurement of elbow flexion and knee extension.^29^ Participants will undertake a six minute walk test (6MWT)^30^ prior to being asked the Life-Space^31^ and QuickDASH (QD)^13^ questionnaires. Finally participants will be provided with an accelerometer,^32^ which they will be asked to wear for one week.

### Participant retention plan

The largest threat to validity in a prospective ICU follow-up study is selection bias through a high loss to follow-up rate, which is a common criticism of these studies.^33^ To counter this, the MSK-ICU study has a participant retention plan based on those used in previous successful studies.^34^ The primary study will follow-up participants at a single time point using a telephone questionnaire. There will be a clear method for the recording of both participant contact information and follow-up. A participant contact retention point will be factored into the ICU follow-up clinic at each centre, which occurs at two to three months post ICU discharge.

### Sample size

#### Primary study: telephone follow-up

The sample size calculation is based on the analysis requiring the largest sample size, which is to identify prognostic factors for the development of a lower MSK-HQ Score at six months after admission to ICU. Based on a local case mix data for the participating ICUs, approximately 1700 admitted patients have an ICU length of stay greater than 48 hours and are discharged to a ward within the hospital annually. Approximately 400 patients would be ineligible for participation in the study, and when accounting for an inpatient mortality of 7%, approximately 1200 eligible patients would be expected to survive to discharge from hospital. For the purpose of developing a prediction model, the MSK-HQ Score will be treated as a continuous variable. There are 15 potential baseline prognostic factors identified. Based on this number of predictors and assuming an approximately normal distribution of residuals, the minimum sample size required to estimate a multiplicative margin of error of 0.1 would be 249 individuals.^35^ Allowing for a 25% loss to follow-up, it is necessary to recruit 332 participants. This sample size and number of predictors would also ensure the estimation of a shrinkage factor ≥0.9 and a difference between apparent and adjusted *R*^2^≤0.02, even with a moderate anticipated *R*^2^ of 0.6.

#### Substudy 1: in-person MSK assessment

A recent review identified that previous ICU follow-up studies, that have included a physical assessment of some aspect of MSK health, have varied in sample size from 11 to 127 (ref). A previous UK-based single-centre prospective cohort study(ref) involving similar physical assessments had a sample size of 61 participants at six months following hospital discharge with an 18-month recruitment period. As the aim of this part of the study is to describe the specific MSK conditions and their prevalence, across three sites the target sample size is 115 participants.

#### Substudy 2: in-person functional assessment

The single previous study to more comprehensively evaluate mobility in ICU survivors included 24 participants.^36^ As the participants will be a subgroup based on MSK health state and the exploratory study involves a greater travel and time commitment, the sample size is likely to be relatively small compared with the other parts of the study. Therefore the target sample size is 35.

### Statistical analysis

#### Primary study: telephone follow-up

Descriptive statistics will be used to examine baseline characteristics and the distribution of individual components will be examined in the population. Descriptive statistics will also be used to examine the results of the questionnaires collected (point estimates and variability), including: MSK-HQ, EQ-5D-5L utility score, employment, HADS and IES-R. Correlation statistics will be used to assess the relationships between MSK-HQ and employment, EQ-5D-5L, HADS and IES-R. Multivariable regression will be used to assess for association of the baseline variables collected with the MSK-HQ score. A detailed statistical analysis plan describing all steps for the model development will be written and made publicly available.

#### Substudy 1: in-person MSK assessment

Descriptive statistics will be used to examine the individual aspects of the MSK assessment (FABQ, ROM, VAS, MRCSS, dynamometry). Correlation statistics will be used to explore the relationships between the individual aspects of assessment and the MSK-HQ Score.

#### Substudy 2: in-person functional assessment

Descriptive statistics will be used to examine the individual components of the assessment (US, IKD, 6MWT, life-space questionnaire, accelerometery, QD). Correlation statistics will be used to explore the relationships between the results at impairment and function domains in the upper and lower limb.

### Ethics and dissemination

#### Ethics

This study received ethical approval from the North of Scotland Research Ethics Committee. The Oxford University Hospitals NHS Foundation Trust will act as sponsor. This paper reports protocol version 4 (December 2022) and has been written with reference to the Strengthening the Reporting of Observational Studies in Epidemiology checklist, which will also be used to report the results of the study.^37^

Participants who are identified as having a new MSK impairment at any of the three potential contact points will be given appropriate self-care advice (which may include attending their General Practitioner [GP] surgery), and have a comprehensive written summary of findings sent to their local ICU follow-up clinic and GP.

The questionnaires being used at the telephone follow-up may have the potential to result in distress to participants. Prior to commencing the questionnaires the researcher will explain that the participant is free to ask to pause or stop at any time, and will offer to stop or pause the questionnaires if the participant becomes distressed during the telephone conversation. Participants will be offered advice on avenues for support such as ICU support groups.

Any clinically concerning information that is reported by participants at any point will be discussed with their GP and appropriate referrals made within the existing hospital system. Patients presenting with a more serious clinical problem will be advised to attend their local Minor Injuries Unit or Emergency Department, or an ambulance will be called as appropriate.

### Dissemination

Results from this study will be disseminated at regional and international conferences and in peer-reviewed journals. Authorship of any papers related to this study will follow the ICMJE recommendations (http://www.icmje.org/recommendations/).

## Supplementary Material

Reviewer comments
