# Supplementary material for: Musculoskeletal health state and physical function of intensive care unit survivors: protocol for a UK multicentre prospective cohort study (the MSK-ICU study)
Source: BMJ Open. 2023 Feb 2;13(2):e071385. doi: 10.1136/bmjopen-2022-071385 (PMC9896246; doi:10.1136/bmjopen-2022-071385)
Supplement: Reviewer comments [file bmjopen-2022-071385.reviewer_comments.pdf]

PEER REVIEW HISTORY

BMJ Open publishes all reviews undertaken for accepted manuscripts. Reviewers are asked to complete a checklist review form (**http://bmjopen.bmj.com/site/about/resources/checklist.pdf**) and are provided with free text boxes to elaborate on their assessment. These free text comments are reproduced below.

ARTICLE DETAILS

**TITLE (PROVISIONAL)**	The musculoskeletal health state and physical function of intensive care unit survivors: protocol for a UK multicentre prospective cohort study (the MSK-ICU study)
**AUTHORS**	Gustafson, Owen; King, Elizabeth; Schlussel, Michael; Rowland, Matthew; Dawes, Helen; Williams, Mark

This article was not externally reviewed at BMJ Open. Protocols that have been independently assessed prior to submission to BMJ Open are usually fast-tracked to publication on the grounds that further substantial changes will not be possible. This independent assessment will usually be external, independent review for both a substantial grant award from a non-commercial or government funder and ethics approval.
